# Unraveling the Evolutionary Tales of *Yunnanopilia longistaminea* (Opiliaceae): Insights from Genetic Diversity, Climate Adaptation, and Conservation Strategies

**DOI:** 10.3390/plants14050706

**Published:** 2025-02-26

**Authors:** Guansong Yang, Liu Yang, Shikang Shen, Yuehua Wang, Yuying Wang

**Affiliations:** 1 College of Landscape and Horticulture, Yunnan Agricultural University, Kunming 650201, China; wyysxp@126.com; 2 College of Ecology and Environmental Science, Yunnan University, Kunming 650500, China; yangliu@ynu.edu.com (L.Y.); yunda123456@126.com (S.S.); wangyh58212@126.com (Y.W.)

**Keywords:** genetic diversity, genetic structure, DNA sequences

## Abstract

The evolutionary history of *Yunnanopilia longistaminea*, a vulnerable plant endemic to the Yuanjiang-Honghe River Valley in southwestern China, was investigated using cpDNA and nrDNA sequences along with ecological niche modeling. Understanding the genetic diversity and population structure of *Y. longistaminea* is crucial for developing effective conservation strategies and managing its genetic resources. This study comprehensively sampled 295 individuals from 16 populations, which represent the species’ entire global distribution range, ensuring a thorough and representative analysis of its genetic diversity and population structure. The results revealed high genetic diversity and population structure, with significant genetic differentiation among populations. Specifically, the total nucleotide diversity was 2.40 × 10^−3^ for cpDNA and 1.51 × 10^−3^ for nrDNA, while the total haplotype diversity was 0.605 for cpDNA and 0.526 for nrDNA. The divergence time of ancestral haplotypes of *Y. longistaminea* was estimated to be around 2.19 million years ago based on nrDNA and 2.72 million years ago based on cpDNA. These divergence times are comparable to those of other ancient plant species, suggesting a long evolutionary history. The population size of *Y. longistaminea* was found to have significantly declined around 30,000 years ago. The current distribution model suggests that *Y. longistaminea* primarily inhabits the warm temperate zone of China, and the LGM distribution model predicts a concentration of the species in Yuanjiang-Honghe River Valley in southwestern China. This study concludes that the southwestern region of China may have served as a glacial refuge for *Y. longistaminea*. These findings suggest that establishing protected areas in these regions and creating gene banks for ex situ conservation could be effective strategies to preserve the genetic diversity of *Y. longistaminea*. Further research on its population dynamics and genetic adaptation to climate change is valuable for understanding the species’ evolutionary history and conservation.

## 1. Introduction

The genetic composition of extant species, particularly long-lived and sedentary organisms, is shaped by historical processes [1]. The genetic structure of many species has been used to infer relationships between historical vicariance and geological changes, dispersal history, and periods of expansion and contraction associated with global climate change [2]. Climate change affects genetic diversity by altering population dynamics [3], and genetic information can reveal the adaptive potential of species in postglacial colonization refugia [4,5]. Assessing genetic diversity at the population level provides valuable insights into the conservation and utilization of germplasm resources, as this variation directly reflects the evolutionary and ecological processes experienced by the species [6]. Given the importance of genetic diversity in shaping species’ responses to environmental changes, understanding the historical and ecological processes that shape this diversity is crucial.

The geographical distribution of plant species has been significantly influenced by climatic oscillations during the Quaternary period [7]. Such climate fluctuations have triggered species colonization or contraction, resulting in genetic subdivision and population admixture [8]. In China, especially in the Qinghai–Tibet Plateau (QTP) and adjacent regions, significant progress has been made in inferring the Quaternary phylogeographic histories of plant species using population genetics approaches [9,10,11,12,13]. Studies have shown that the QTP acted as a barrier against glaciation within the warm temperate zones of China, resulting in arid climates that persisted for thousands of years [14,15,16,17,18,19]. Thus, the present warm temperate region likely served as glacial refugia for plant species during the Last Glacial Maximum (LGM), approximately 23,000 to 18,000 years ago. This hypothesis is further validated through phylogeographic studies, which provide indirect evidence for the timing and location of these refugia [20]. However, it remains unclear whether the genetic differentiation of plant populations within these regions is due to isolation on a heterogeneous landscape or adaptation along ecological gradients. This region’s unique geological and climatic history has shaped its diverse topography and microclimates, influencing species distribution and genetic structure. The complex interplay of these factors makes it an ideal case study area for examining the long-term impacts of environmental changes on biodiversity.

Southwest China (including Sichuan, Yunnan, Guizhou, Tibet, and Chongqing) is a key area for studying plant responses to past climate changes due to its complex topography and diverse climates [21]. Despite extensive research in this region, studies focusing on species endemic to the Yuanjiang-Honghe Valley remain relatively scarce, likely due to the valley’s unique dry-hot climate, fragile ecosystem, and limited accessibility, which pose significant challenges for field research. The river originates in northwest Yunnan, China, and flows through southwest Yunnan and northern Vietnam before emptying into the Gulf of Tonkin [22]. The Yuanjiang-Honghe River basin is connected to the Red River Fault Zone (RRFZ), which was formed due to the uplift of the Himalayas and the expansion of the South China Sea basin. Spanning over 1000 km on land, the RRFZ constitutes a significant geological fault zone in Yunnan that sustains a diverse array of broad-leaved tree species, many of which are endemic to this region [23]. Focusing on *Y. longistaminea*, an endangered and endemic species, this study addresses a critical gap in our knowledge about the evolutionary and conservation status of this unique plant, particularly during the Quaternary period when significant climatic and geological changes occurred.

This study focuses on *Y. longistaminea* (W. Z. Li) C. Y. Wu and D. Z. Li (Figure S1), a monotypic species in the Opiliaceae family and an endangered plant endemic to the Yuanjiang-Honghe River Valley in southwestern Yunnan, China. The species is under serious threat due to overexploitation of wild vegetables, as well as habitat destruction caused by agricultural expansion and infrastructure development, with many populations in steep decline. *Yunnanopilia* is the most primitive genus in the Opiliaceae family. Based on our previous findings, the plant is primarily distributed along the banks of rivers in Southwest China and Southeast Asia. Its large seeds have limited dispersal ranges and are susceptible to desiccation [24,25]. Consequently, inbreeding is prevalent within this species, leading to high levels of genetic differentiation and structure, as evidenced by maternal genetic DNA analyses [26]. Despite its significance as a germplasm resource and an endangered species, the genetic diversity and population structure of *Y. longistaminea* remain largely unexplored, and the causes of its population decline and endangerment are still unclear. Field surveys have revealed that many populations are facing severe threats, primarily due to overexploitation of wild vegetable resources, as well as habitat destruction caused by agricultural expansion and infrastructure development over the past few decades. Therefore, there is an urgent need to conduct a comprehensive assessment of its genetic diversity and population structure to formulate effective conservation strategies and germplasm utilization methods. Our findings will not only enhance our understanding of *Y. longistaminea*’s evolutionary history but also provide critical insights into its conservation. We plan to use these insights to establish protected areas in regions with high genetic diversity, create gene banks for ex situ conservation, and conduct further research on population dynamics and genetic adaptation to climate change, aligning with the significant genetic differentiation and demographic changes observed.

Building on this understanding, our study aims to conduct a phylogeographical analysis of *Y. longistaminea* by tracing variations in four chloroplast intergenic spacers (cpDNA) and one nuclear sequence (nrDNA) across 13 populations. We aimed to test the following hypotheses: (i) local environmental variations in its habitats have influenced the lineage divergence of *Y. longistaminea*; (ii) climate changes, particularly the glacial–interglacial cycles of the Quaternary, have reshaped the species’ genetic structure and accelerated its diversification. The innovations of this study include (1) the first systematic assessment of the genetic diversity and population structure of *Y. longistaminea* and (2) the exploration of its response mechanisms to climate change, providing new insights into its evolutionary history and a scientific basis for the conservation of this endangered species.

## 2. Materials and Methods

### 2.1. Population Sampling

**Figure 1:**
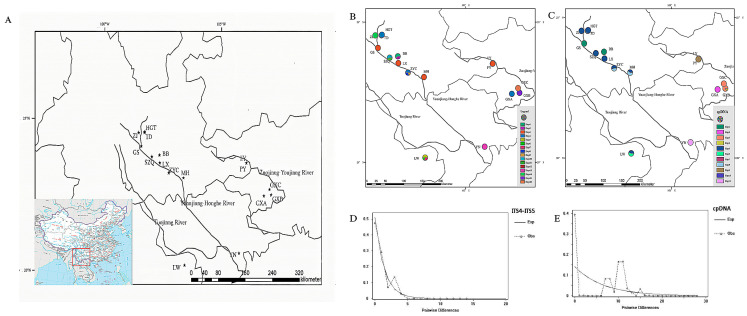
Geographic distribution map of the population of *Yunnanopilia longistaminea* (**A**). The haplotypes and genotypes based on nuDNA (**B**) and cpDNA (**C**). Multimodality mismatch distribution curves of nuDNA and cpDNA in the overall populations are shown in (**D**,**E**). Note: Asterisks indicate the location of the population sampled.

For this study, leaf samples of 295 individuals of *Y. longistaminea* were collected from 16 natural populations, representing the species’ entire geographic distribution range within the warm temperate zone of China (see Figure 1A and Table 1). Eight to 20 individuals were sampled for each population, with all individuals being at least 15 m apart. The populations were distributed across Yunnan Province (11 populations), Guangxi, China (3 populations), Laos (1 population), and Vietnam (1 population). Voucher specimens were obtained for each population and deposited at the Herbarium of the College of Horticulture and Landscape, Yunnan Agricultural University, Kunming, Yunnan, China. The latitude, longitude, and altitude of each population were recorded using a handheld GPS device (Garmin International eTrex 30x, Inc., Olathe, KS, USA) with an accuracy of ±5 m.

### 2.2. Molecular Procedures

Total genomic DNA was isolated from young and healthy leaves using the CTAB procedure [27]. This study chose four cpDNA intergenic spacers and one nrDNA internal transcribed spacer for full analysis: *atp*B-*rbc*L [28], *trn*L-*trn*F [29], *psb*A-*trn*H [30], *trn*G-*trn*S [31], and ITS4-ITS5 [32]. ITS4-ITS5: TCCTCCGCTTATTGATATGC, ITS5: GGAAGTAAAAGTCGTAACAAGG. psbAF: GTTATGCATGAACGTAATGCTC, *trn*HR: CGCGCATGGTGGATTCACAAATC *atp*B: ACATCKARTACKGGACCAATAA, *rbc*L: AACACCAGCTTTRAATCCAA, *trn*L; CGAAATCGGTAGACGCTACG, *trn*F: ATTTGAACTGGTGACACGAG *trn*G: GAACGAATCACACTTTTACCAC, *trn*S: GCCGCTTTAGTCCACTCAGC. The polymerase chain reaction (PCR) contained 2.0 μL of template DNA, 1.0 μL of dNTPs, 2.5 μL of 10 × PCR buffer (containing MgCl_2_), 0.3 μL of the primer, 0.3 μL of Taq DNA polymerase (Takara, Shiga, Japan), and 13.6 μL of double-distilled water in a volume of 20 μL. The conditions of the PCR amplifications were as follows: 80 °C for 5 min, followed by 29 cycles consisting of 1 min at 95 °C, 30 s at 50 °C (cpDNA sequences), 1.5 min at 65 °C; and a final step of 9 min at 72 °C (Supplementary Tables S1–S3). All PCR products were used with an ABI 3770 automated sequencer.

### 2.3. Data Analysis

All sequences were visualized and edited, and then implemented in Seq Man. The sequences were conducted for multiple alignments using Clustal X, version 1.83 [33] and adjusted in Bioedit, version 7.0.4.1 [34]. After four cpDNA regions were combined. A congruency test for the four combined cpDNA regions showed a significant rate of homogeneity (*p* > 0.5) in PAUP* 4.0b10 [35], suggesting a high degree of homogeneity between the two cpDNA regions. The combined cpDNA sequences were therefore used in the following analysis.

Haplotypes were calculated from aligned DNA sequences in Dna SP, version 5.0 [36]. Within and among-population genetic diversities were estimated by calculating Nei’s nucleotide diversity (Pi) and haplotype diversity (Hd) indices using Dna SP, version 5.0 [36]. We calculated the within-population gene diversity (HS); the gene diversity in total populations (HT = HS + DST, DST, gene diversity between populations) [37]; and two measures of population differentiation, GST and NST, according to the methods described by Pons and Petit using Permut Matrix software (version 1.9.4) [38]. We compared GST and NST using the U-statistic, which is approximated by a Gaussian variable by taking into account the covariance between GST and NST, and a one-sided test. When NST is larger than GST, the phylogeographic structure is obvious, which indicates that closely related haplotypes were found more often in the same area than less closely related haplotypes [38].

We used the program Arlequin, version 3.11, to conduct an analysis of molecular variance (AMOVA) and to estimate the genetic variation that was assigned within and among populations [38,39]. Phylogenetic relationships among cpDNA and nrDNA haplotypes of *Y. longistaminea* were inferred using maximum parsimony (MP) in PAUP* 4.0b10 [35] and Bayesian methods implemented in MrBayes, version 3.1.2 [40]. *Champereia manillana* was used as the outgroup. We used Mega version 5.0 [41] to construct a neighbor-joining (NJ) tree that was based on the neighbor-joining method without using an outgroup. The degree of relatedness among cpDNA and among nrDNA haplotypes was also estimated using Network, version 4.2.0.1 [42]. In the network analysis, indels were treated as single mutational events.

### 2.4. Ecological Niche Modeling

In this study, the Maxent version was used to conduct ecological niche modeling for *Y. longistaminea* and to compare the distributions during the Last Glacial Maximum (LGM) [43]. This study obtained the geocoordinates of 35 occurrence data of *Y. longistaminea*. All data come from the Chinese Virtual Herbarium (CVH, https://www.cvh.ac.cn/, accessed on 25 January 2024), Global Biodiversity Information Facility (http://www.gbif.org/, accessed on 25 January 2024), and bioclimatic variables from the World Clim database (https://www.worldclim.org, accessed on 25 January 2024). The data employed 20 replicates based on 80% of the distribution coordinates for training and 20% for testing and adopted the model with the best AUC values [43]. This study performed a jackknife test to estimate the percent contributions of bioclimatic variables to the prediction for the distributional models. This study also employed the “10 percentile presence” threshold logistic approach as determined by Maxent in order to distinguish the threshold between suitable and unsuitable habitats for further analyses. This study drew Graphics for each predicted SDM using DIVA-GIS 7.5.

## 3. Results

### 3.1. Genetic Diversity and Structure

The length of chloroplast DNA (cpDNA) sequences, including atpB-rbcL, trnL-trnF, psbAF-trnHR, and trnG-trnS, ranged from 384 to 864 base pairs (bp). These sequences were aligned to a common length of 2595 bp, revealing 48 polymorphic positions and 20 indels (Supplementary Tables S4–S8). The analysis identified 11 distinct haplotypes (H1–H11) based on combined cpDNA data (Figure 1C). The total length of nuclear ribosomal DNA (nrDNA) sequences (ITS4-ITS5) was 638 bp, with 11 polymorphic positions and 3 indels (Supplementary Table S9). Sixteen chloroplast haplotypes (H1–H16) were identified (Figure 1B).

The genetic diversity analysis of four combined chloroplast DNA (cpDNA) fragments in *Y. longistaminea* revealed 16 cpDNA haplotypes with a haplotype diversity (*H*d) of 0.605 and a nucleotide diversity (*P*i) of 0.00241 (Table 2). The overall genetic diversity of *Y. longistaminea* was high (*H*_T_ = 0.953), significantly exceeding the average within-population genetic diversity (*H*_S_ = 0.330) (Table 3). At the population level, the nucleotide and haplotype diversities were as follows: for the LY population in Yunnan (*H*d = 0.20, *P*i = 0.00016) and the GXB population in Guangxi (*H*d = 0.333, *P*i = 0.00013) (Table 2). The analysis at the population level showed high genetic differentiation among populations, with *G*_ST_ = 0.949 and *N*_ST_ = 0.983, indicating that the genetic diversity among populations was much higher than that within populations (Table 3). The U-test showed that *N*_ST_ was greater than *G*_ST_, but the difference was not statistically significant (*p* > 0.05).

The genetic diversity analysis of *Y. longistaminea* based on nrDNA (ITS4-ITS5) revealed a nucleotide diversity (*P*i) of 0.00151 and a haplotype diversity (*H*d) of 0.526 (Table 2). The overall genetic diversity of *Y. longistaminea* was high (*H*_T_ = 0.883), significantly exceeding the average within-population genetic diversity (*H*_S_ = 0.203) (Table 3). The nucleotide and haplotype diversities within populations were as follows: The LW population (*H*d = 0.6889, *H*d = 0.00144), LX population in Yunnan (*H*d = 0.6889, *P*i = 0.00013), SZQ population in Yunnan, and TD population in Yunnan (*H*d = 0.5333, *P*i = 0.00084) (Table 3). At the population level, genetic differentiation was observed among populations, with *G*_ST_ = 0.651 and *N*_ST_ = 0.834. The U-test showed that *N*_ST_ was greater than *G*_ST_, indicating significant phylogeographic structure (*p* > 0.05).

The AMOVA revealed that 98.50% of the genetic variation was partitioned among populations and 1.50% was within populations at the cpDNA level (Table 4). The AMOVA revealed that 87.60% of the genetic variation was partitioned among populations and 12.40% was within populations at the nrDNA level (Table 4). The results showed that *Y. longistaminea* has a high level of genetic variation among populations and, as a result, has a high population structure (Table 4).

### 3.2. Evolutionary Network Analysis

**Figure 2:**
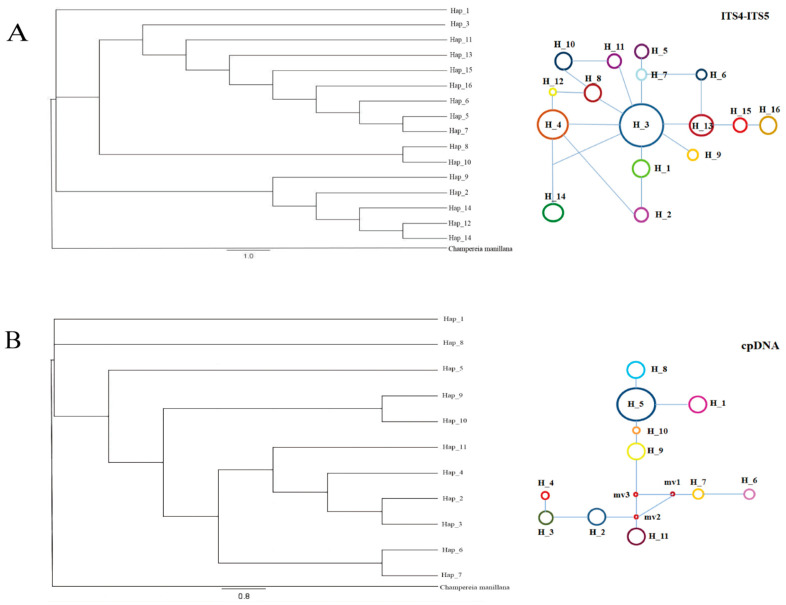
Phylogenetic trees of *Yunnanopilia longistaminea* calculated by Bayesian inference (BI) of (**A**) nuDNA and (**B**) cpDNA. Numbers above the branches indicate the bootstrap 1000. Note: The size of the circle corresponds to the frequency of each haplotype, different colors correspond to different haplotypes, and the small red circle represents a mutation step.

The phylogeny of cpDNA and nrDNA haplotypes was constructed using the maximum resolution (MP) and Bayesian methods. *C. manillana* was used as an outgroup. Haplotypes derived from both chloroplast DNA (cpDNA) and nuclear ribosomal DNA (nrDNA) sequences (ITS4-ITS5) were utilized in the construction of reticulated branching diagrams for 16 populations of *Y. longistamineas* (Figure 2). The haplotype maps generated from ITS4-ITS5 sequences revealed that Haplotypes 4 and 3 were situated in the central region of the reticulation branch with a high frequency, while haplotypes located at the periphery of the branch exhibited low frequency. Additionally, it was observed that several haplotypes descended from the ancestral haplotypes Hap 4 and Hap 3 (Figure 2A).

In the reticulated branching diagram constructed from cpDNA, the haplotypes were partitioned into upper and lower branches, with putative haplotype deletions inferred between the two branches, indicating the potential existence of an intermediary population between Hap 9 and Hap 11 in the past. Utilizing maximum parsimony and Bayesian methods, a phylogenetic tree was constructed based on haplotypes derived from both chloroplast and nrDNA fragments. The results indicated a substantial concordance between the topological structure of the phylogenetic tree and the reticulated evolutionary map. The neighbor-joining tree constructed from nrDNA, Hap 1 formed a distinct branch, while the remaining haplotypes could be categorized into three separate branches. Conversely, in the phylogenetic tree constructed from cpDNA, Hap 1 and Hap 8 constituted a single branch; Hap 11, Hap 4, Hap 2, and Hap 3 clustered together in a single branch; Hap 9 and Hap 10 formed a group; and Hap 6 and Hap 7 were grouped together (Figure 2B).

### 3.3. Phylogeography and Divergence Time

**Figure 3:**
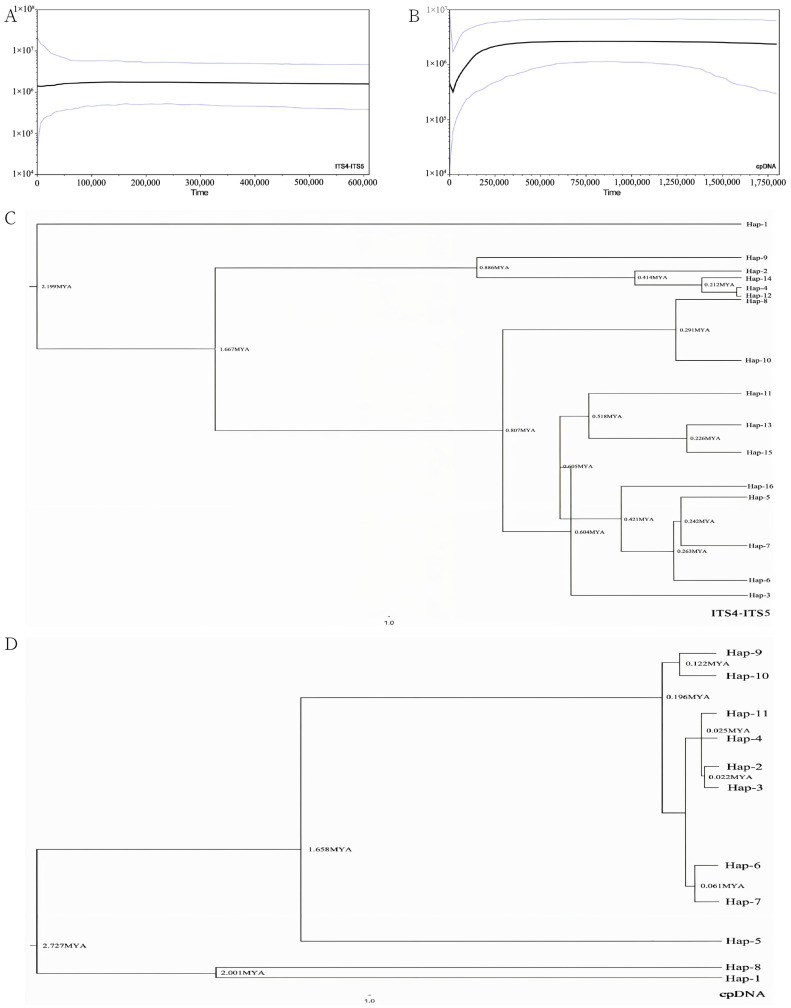
Bayesian skyline plot based on nrDNA (**A**) and cpDNA (**B**) for the effective population size fluctuation throughout time. Neighbor-joining and Bayesian trees were built by using genetic distance based on nrDNA (**C**) and cpDNA (**D**) haplotypes. Note: The black line is the median estimate; The area between gray lines is 95% confidence interval.

Utilizing maximum parsimony, reticulate branching, and principles of phylogenetic inference, coupled with the application of BEAST software (v10.X, https://beast-dev.github.io/beast-mcmc, accessed on 25 January 2024) [26], our analyses indicate that the divergence time of ancestral haplotypes of *Y. longistaminea* occurred approximately 2.199 million years ago (MYA) based on nrDNA (ITS4-ITS5) haplotype calculations (Figure 3C), and approximately 2.727 million years ago (MYA) based on a cpDNA combined haplotype analysis (Figure 3D). Specifically, the earliest divergence was observed in cpDNA haplotype 1 and haplotype 8 (Hap-1 and Hap-8), estimated to have occurred around 2.001 MYA (Figure 3D). Furthermore, the clade comprising Hap 2, 3, 4, 6, 7, 9, 10, 11, and 5, and the clade comprising Hap 5, diverged approximately 1.658 MYA (Figure 3D). In the phylogenetic tree constructed from nrDNA, Hap 1 was identified as the earliest diverging haplotype, estimated to have diverged 2.199 MYA (Figure 3C). Additionally, the clade comprising Hap 2, 4, 8, 9, 12, and 14 and the clade comprising Hap 3, 5, 6, 7, 8, 10, 11, 13, 15, 16, diverged approximately 1.667 MYA (Figure 3C).

Based on the cpDNA fragment association data and nrDNA, the outcomes of Tajima’s D and Fu’s *F*s tests, as well as the neutrality test, revealed non-significant positive values for Tajima’s D and Fu’s *F*s in the cpDNA association analysis. The mismatch analysis graph indicated a deviation of observed values from expected values, displaying multiple peaks that were inconsistent with the population expansion model, thereby refuting the hypothesis of population expansion (Figure 3A,B). Conversely, nrDNA exhibited negative values for both Tajima’s D and Fu’s *F*s (Table 5). The mismatch analysis graph for nrDNA indicated a close alignment of observed and expected values, with a single peak in the mismatch curve, consistent with the population expansion model.

Utilizing the BEAST software, a Bayesian skyline plot analysis was conducted on nrDNA (ITS4-ITS5) and cpDNA of *Y. longistaminea*, while considering the evolutionary rates of chloroplasts and plant genes. The ITS4-ITS5 results indicated a significant decline in the *Y. longistaminea* population around 30,000 years ago, followed by stability. Simultaneously, the combined cpDNA results also demonstrated a marked decrease in population size 30,000 years ago (Figure 3B). Additionally, the joint cpDNA outcomes revealed a substantial contraction in population size before 30,000 years ago, followed by a subsequent rebound (Figure 3B, Table 5).

### 3.4. Ecological Niche Modeling

**Figure 4:**
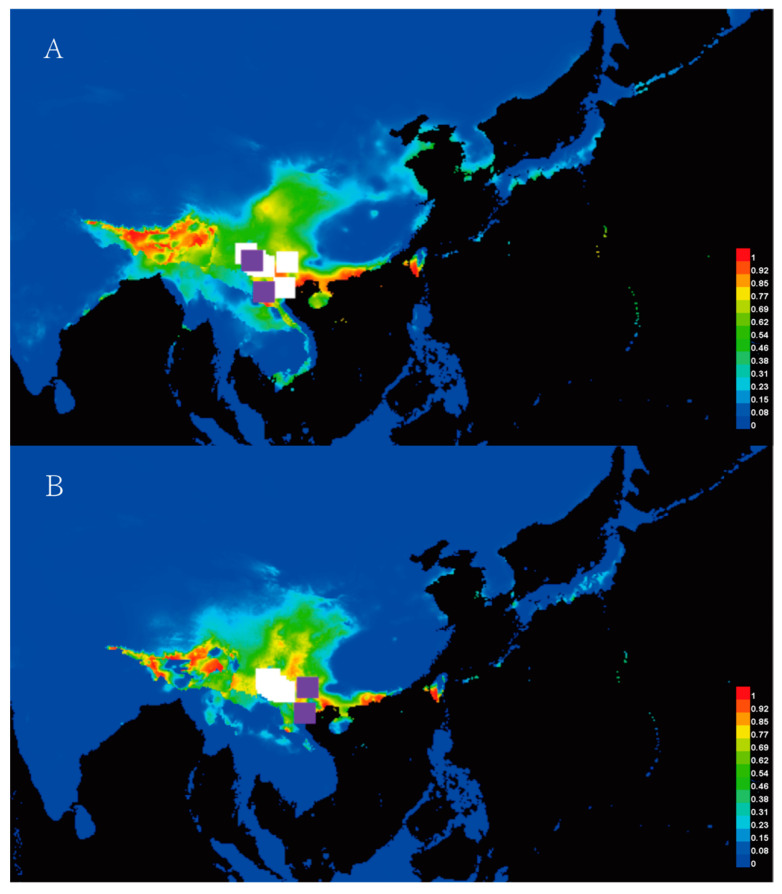
Maps showing the potential distribution by ENM. (**A**) The present. (**B**) Last Glacial Maximum. Note: Different color blocks are different population positions.

The distribution patterns of *Y. longistaminea* during the Last Glacial Maximum (LGM) and the present are depicted in Figure 4. The AUC values, derived from both training and test presence data for the current and LGM periods, exceeded expectations, indicating strong model performance. Notably, the current distribution model suggests that *Y. longistaminea* primarily inhabits the warm temperate zone of China, implying a similar occurrence during the LGM period (Figure 4A). In contrast, the LGM distribution model predicts a concentration of the species in Yunnan and western China, including Sichuan, Tibet, and Laos during the LGM period, with slight decreases in these areas (Figure 4B).

## 4. Discussion

The process of species adapting to their environment in natural conditions involves the continual generation of genetic variations to ensure the population’s reproduction [44,45]. Genetic variation serves as the driving force of evolution and forms the basis for the survival and development of populations. The genetic diversity and population structure of *Y. longistaminea*, an endangered plant endemic to the Red River Valley in southwestern China, provide crucial insights into its evolutionary history and conservation needs. Our study reveals high genetic diversity and significant population structure within this species, with most genetic variation partitioned among populations rather than within them (cpDNA: 98.5% among populations; nrDNA: 87.6% among populations). This pattern is consistent with other endangered plants, such as *Dysosma versipellis* (total nucleotide diversity of 0.0014) and *Munronia delavayi* (total nucleotide diversity of 0.0011) [46,47], which also exhibit high genetic differentiation among populations. However, the haplotype diversity (*H*d = 0.605) of the *Y. longistaminea* was lower than that of other endangered plants, such as *Ligularia hodgsonii* (haplotype diversity of 0.895) [48], *Dysosma versipellis* (haplotype diversity of 0.924) [46], *Cycas simplicipinna* (haplotype diversity of 0.846) [26], *Solanum pimpinellifolium*, *Citrullus colocynthis*, *Tsuga dumosa*, and *Ginkgo biloba* [49,50,51,52]. The limited gene flow among Y. longistaminea populations (Table 3) indicates high isolation, likely driven by restricted seed dispersal and habitat fragmentation. This pattern mirrors that observed in *Sophora davidii*, where genetic diversity is similarly constrained by limited gene flow and human activities [44,45].

It is worth noting that the geographic distribution of cpDNA haplotypes is notably distinct from that of nuDNA genotypes (Figure 2). Such discordance between organellar and nuclear DNA has been documented in other species, including *Sophora davidii* [53], *Cycas diannanensis* [1], and *Osteomeles schwerinae* [54]. This discordance between organellar and nuclear DNA may be associated with seed desiccation tolerance. Seed desiccation tolerance is an adaptive trait that has evolved in plants to facilitate survival and reproduction [55]. However, if seeds are intolerant to desiccation, their dispersal range may be restricted, thereby influencing gene flow and genetic diversity. This is supported by our previous findings that seeds of *Y. longistaminea* are desiccation-sensitive [25].

The evolutionary history of *Y. longistaminea* is marked by significant climatic and geological events. The divergence time of ancestral haplotypes dates back to approximately 2.199 million years ago (MYA) based on nrDNA (Figure 3C) and 2.727 MYA based on cpDNA (Figure 3D). These divergence times are comparable to those of other ancient plant species in the Yunnan region, such as *Ceratotropis* (3.62 MYA) [56] and *Stuckenia filiformis* (3.93 MYA) [57]. These early divergence events are closely associated with the uplift of the Qinghai–Tibet Plateau (QTP), but their timing is more recent than the last phase of the QTP uplift [58]. Therefore, we propose that the geographical isolation of *Y. longistaminea* may have been driven by the late Pliocene uplift of the QTP. Furthermore, the high genetic diversity and the presence of unique haplotypes in the southwestern region of China suggest that this area may have served as a glacial refuge during the Last Glacial Maximum (LGM). This hypothesis is supported by ecological niche modeling, which predicts a concentration of *Y. longistaminea* in Yunnan and central China during the LGM (Figure 4B). In contrast, other plant species in Yunnan, such as *Rhododendron chinensis*, have experienced diversification from the Pliocene to the Pleistocene under the favorable climate of the temperate and subtropical zones [59]. However, the relatively recent divergence times of *Y. longistaminea* indicate that its diversification may be more sensitive to recent climatic fluctuations. The seeds of *Y. longistaminea* exhibit physiological epicotyl dormancy, a phenomenon also observed in other species such as *Humboldtia laurifolia* [60]. This dormancy mechanism likely enables the species to adapt to seasonal climatic changes and survive through glacial periods. Moreover, the responsiveness of its seeds to stratification and gibberellic acid (GA_3_) suggests that dormancy release is closely linked to environmental cues. These adaptive traits may have played a crucial role in the species’ survival during climatic oscillations between glacial and interglacial periods, further shaping its population dynamics and distribution patterns.

The population dynamics of *Y. longistaminea* reflect its adaptation to climatic changes during the Last Glacial Maximum (LGM, approximately 26,000 to 20,000 cal BC) and subsequent post-glacial expansion. The Bayesian skyline plot analysis indicates a significant population decline around 30,000 years ago, which is slightly earlier than the commonly accepted timeframe for the LGM (Figure 3). This early decline may be due to regional climatic variations or the specific ecological requirements of the species, leading to the contraction of suitable habitats. However, the species appears to have survived in situ, as evidenced by the negative Tajima’s D and Fu’s Fs values in nrDNA (Table 5), which suggest subsequent population expansion events. The star-like pattern of the haplotype network further supports the hypothesis of rapid population expansion following the LGM (Figure 2). An analysis of the cpDNA haplotype network reveals a pattern of continuous haplotype loss between Hap9, Hap11, Hap2, and Hap7, with these haplotypes gradually diverging into four distinct branches. This pattern is likely associated with the onset of the glacial period (Figure 2). The limited dispersal ability of *Y. longistaminea* seeds and its preference for evergreen broad-leaved forests have likely contributed to its fragmented distribution and high genetic differentiation among populations (Table 4). This pattern is consistent with other plant species in China that survived the LGM in situ, such as *Platycarya strobilacea*, *Cercidiphyllum japonicum*, and *Cotinus coggygria* [61,62,63]. These species were able to persist in localized refugia during the glaciation period, primarily in warm temperate regions dominated by evergreen or temperate deciduous forests, such as the Yuanjiang-Honghe River area in Yunnan. Similar to these species, *Y. longistaminea* may have occupied local glacial refugia in these regions, where it was protected from the full impact of the ice age by topographical barriers such as the Qing–Tibet Plateau. This is supported by the combined analysis of cpDNA and the composition and distribution of nrDNA haplotypes in *Y. longistaminea*, as well as the analysis of nucleotide diversity parameters (Table 2), which show that the cpDNA (LW, MH, LX) and nrDNA (LW, LX, TD) with higher haplotype diversity and nucleotide diversity further confirm this. In contrast, plant species with broader ecological tolerances or higher dispersal abilities may have experienced more extensive range shifts during and after the Last Glacial Maximum (LGM). For example, studies on *Haloxylon ammodendron* and *Haloxylon persicum* have shown significant range contractions during the Last Glacial Maximum (LGM) due to arid conditions and limited water availability. These species were able to persist in localized refugia, primarily in regions with access to groundwater, such as the Gurbantunggut Desert in Central Asia [64]. Post-glacially, they expanded rapidly through key migration corridors, facilitated by their ecological traits, such as drought tolerance and efficient water uptake. This highlights the importance of both ecological traits and historical climatic events in shaping the current distribution and genetic structure of plant species. Additionally, in the Yuanjiang-Honghe River area, species such as *Broussonetia papyrifera* and *Pistacia weinmanniifolia* have also demonstrated similar patterns of range contraction and expansion in response to climatic changes [65,66]. These species were able to persist in localized refugia during the LGM and subsequently expanded their ranges as climatic conditions improved. The phylogeographic structure of *Y. longistaminea* is consistent with the hypothesis that it survived in situ during the LGM and subsequently expanded its range. Specifically, the Bayesian skyline plot analysis indicates a significant population decline around 30,000 years ago, coinciding with the LGM, and a subsequent population expansion event around 20,000 years ago as climatic conditions improved. Its limited dispersal ability and habitat preference contributed to its fragmented distribution and high genetic differentiation. Future studies should further explore the ecological and genetic mechanisms underlying its survival and expansion, especially in the context of ongoing climate change.

The findings of this study have significant implications for the conservation of *Y. longistaminea*. The high genetic diversity and unique haplotypes in the southwestern region of China highlight the importance of protecting this area as a glacial refuge. Establishing protected areas and gene banks for ex situ conservation could be effective strategies to preserve the genetic diversity of this endangered species [23]. Additionally, further research on the population dynamics and genetic adaptation of *Y. longistaminea* to climate change will provide valuable insights into its evolutionary history and inform conservation efforts. The genetic diversity, population structure, and evolutionary history of *Y. longistaminea* reflect its adaptation to climatic and geological changes in the southwestern region of China. The high genetic differentiation among populations and the presence of unique haplotypes in the southwestern region indicate that this area may have served as a glacial refuge for the species. Conservation efforts should focus on protecting these regions and preserving the genetic diversity of *Y. longistaminea* to ensure its survival in the face of ongoing environmental changes. While our study provides valuable insights, it is limited to the analysis of cpDNA and nrDNA markers and does not consider other potential influencing factors.

## 5. Conclusions

We investigated the evolutionary history of *Y. longistaminea* using cpDNA and nrDNA sequences and ecological niche modeling. The genetic data revealed high differentiation among populations, with distinct lineages corresponding to different geographic regions. The divergence of ancestral haplotypes occurred around 2.199 million years ago (MYA) based on nrDNA and 2.727 MYA based on cpDNA. The ENM results showed a concentration of potential distributions in Yunnan and central China during the Last Glacial Maximum (LGM), with a slight expansion in the current period. These findings highlight the importance of protecting the genetic diversity of *Y. longistaminea* through conservation measures such as establishing protected areas and gene banks. Future research on population dynamics and genetic adaptation will provide further insights into its evolutionary history and inform effective conservation strategies.

## Figures and Tables

**Table 1:** Sampling of *Yunnanopilia longistaminea* in the present study.

Code	Populations	Location	Longitude (E°)	Latitude (N°)	Altitude (m)	N.
P1	GS	Gasa town, Yunnan province	101.5574	24.0793	1389	6
P2	SZQ	Qinglongchan town, Yunnan province	102.0168	23.7291	1311	57
P3	BB	Baoxiu town, Yunnan province	102.3381	23.7802	1390	27
P4	ZYC	Niujie town, Yunnan province	102.3572	23.5371	1217	20
P5	LY	Daleng town, Yunnan province	106.0697	23.5337	736	15
P6	PY	Gula town, Yunnan province	106.0581	23.5169	826	15
P7	YN	Hoa Binh province, Vietnam	105.7467	20.5561	527	15
P8	LW	Houphan province, Laos	103.4102	20.1666	1100	15
P9	MH	Manhao town, Yunnan province	103.3642	23.0406	783	15
P10	LX	Majie town, Yunnan province	102.7385	23.1983	960	20
P11	ZJ	Fabiao town, Yunnan province	101.4518	24.5271	1490	20
P12	TD	Tuodian town, Yunnan province	101.6970	24.5320	1463	20
P13	HGT	Dutian town, Yunnan province	101.7147	24.5494	1507	20
P14	GXA	Shanglong town, Guangxi province	106.8130	22.4414	360	10
P15	GXB	Xiangshui town, Guangxi province	107.117	22.4769	336	10
P16	GXC	Leiping town, Guangxi province	107.058	22.6446	407	10
Total						295

**Table 2:** The composition of haplotypes, haplotype diversity (*H*d), and nucleotide diversity (*P*i) surveyed for combined cpDNA sequences and nrDNA sequences of *Y. longistaminea* investigated in this study.

Population Code		cpDNA			nrDNA		
		Haplotyoe	*H*d	*P*i	Haplotyoe	*H*d	*P*i
1	GS	Hap 1(4)	0	0	Hap 3(4)	0	0
2	SZQ	Hap 5(9)	0	0	Hap 10(5) Hap11(4)	0.556	0.00088
3	BB	Hap 1(8)	0	0	Hap 1(6) Hap(4)	0	0
4	ZYC	Hap 5(10)	0	0	Hap 3(10)	0	0
5	LY	Hap 9(9)	0.2	0.00016	Hap 3(10)	0	0
6	PY	Hap 9(6)	0	0	Hap 3(6)	0	0
7	YN	Hap 11(9)	0	0	Hap13(10)	0	0
8	LW	Hap 6(5)Hap10(4)	0	0	Hap 5(5) Hap6(2) Hap7(3)	0.688	0.00144
9	MH	Hap 5(6) Hap 8(4)	0	0	Hap 3(10)	0	0
10	LX	Hap5(5) Hap8(4)	0	0	Hap 3(5) Hap8(3) Hap9(2)	0.688	0.00013
11	ZJ	Hap5(8)	0	0	Hap 14(9)	0	0
12	TD	Hap5(10)	0	0	Hap3(2) Hap4(4) Hap8(3)	0.533	0.00084
13	HGT	Hap5(10)	0	0	Hap 4(10)	0	0
14	GXA	Hap2(3)	0	0	Hap 4(5)	0	0
15	GXB	Hap3(5) Hap4(1)	0	0	Hap 15(4)	0	0
16	GXC	Hap(10)	0	0	Hap 16(5)	0	0
total			0.605	0.0024		0.526	0.00151

**Table 3:** Genetic diversity and differentiation parameters for the combined cpDNA sequences and nrDNA (ITS4-ITS5) sequences in all populations of *Y. longistaminea*.

Markers	*H*_S_	*H*_T_	*G*_ST_	*N*_ST_
cpDNA	0.330	0.953	0.949	0.983
nrDNA	0.203	0.883	0.651	0.833

**Table 4:** Results of the analysis of molecular variance (AMOVA) of the combined cpDNA sequences and nrDNA sequence data from populations of *Y. longistaminea*.

Markes	Source of Variation	d.f.	Sum of Squares	Percentage of Variation (%)	*F*_ST_
cpDNA	Among populations	15	1701.294	98.5%	0.985
	Within populations	116	24.767	1.50%	0.015
nrDNA	Among populations	14	103.915	87.62%	0.876
	Within populations	106	13.622	12.38%	0.124

Notes: d.f freedom.

**Table 5:** Parameters of neutrality tests and demographic analysis based on cpDNA and nrDNA of *Y. longistaminea*.

Markers	Tajima’ *D*	Fu and Li’ *D* *	Fu and Li’ *F* *	Fu’ *F*s
cpDNA	0.992	1.563	1.384	0.00
ITS4-ITS5	−0.570	1.172	0.682	−1.919

Note: * is *p* < 0.05, significant difference.

## Data Availability

Data is contained within the article or Supplementary Materials.

## Supplementary Materials

The following supporting information can be downloaded at https://www.mdpi.com/article/10.3390/plants14050706/s1.
