# Supplementary material for: Unraveling the Evolutionary Tales of Yunnanopilia longistaminea (Opiliaceae): Insights from Genetic Diversity, Climate Adaptation, and Conservation Strategies
Source: Plants (Basel). 2025 Feb 26;14(5):706. doi: 10.3390/plants14050706 (PMC11901472; doi:10.3390/plants14050706)
Supplement: Supplementary file 1 [file plants-14-00706-s001.zip › Supplementary Table.pdf]

Table S1. Information of primers

Primers	Sequence	Reference
ITS4-ITS5	ITS4:TCCTCCGCTTATTGATATGC	White. et al，1990
	ITS5:GGAAGTAAAAGTCGTAACAAGG	
*psb*AF-*trn*HR	psbAF:GTTATGCATGAACGTAATGCTC	Chiang. et al，1998
	*trn*HR:CGCGCATGGTGGATTCACAAATC	
*atp*B-*rbc*L	*atp*B: ACATCKARTACKGGACCAATAA	Fritsch. et al，2001
	*rbc*L：AACACCAGCTTTRAATCCAA	
*trn*L-*trn*F	*trn*L：CGAAATCGGTAGACGCTACG	Sudarmono. et al，2007
	*trn*F：ATTTGAACTGGTGACACGAG	
*trn*G-*trn*S	*trn*G: GAACGAATCACACTTTTACCAC	Shaw. et al，2005
	*trn*S: GCCGCTTTAGTCCACTCAGC	

Table S2. PCR reaction system

Component	ITS4-ITS5(μl)	*psb*AF-*trn*HR(μl)	*atp*B-*rbc*L(μl)	*trn*L-*trn*F(μl)	*trn*G-*trn*S(μl)
体系	20	20	20	20	20
Double-distilled water	12.3	13	13	13	13
PCR buffer	2.0	2.0	2.0	2.0	2.0
$MgCl_2$	1.2	1.0	1.0	1.0	1.0
dNTP	1.0	1.0	1.0	1.0	1.0
DMSO	1.0	1.0	1.0	1.0	1.0
Primer1	0.35	0.3	0.3	0.3	0.3
Primer2	0.35	0.3	0.3	0.3	0.3
Taq DNA 酶	0.35	0.4	0.4	0.4	0.4
Template DNA	2.0	1.0	1.0	1.0	1.0

Table S3. PCR reaction program

*psb*AF-*trn*HR、*atp*B-*rbc*L、*trn*L-*trn*F、*trn*G-*trn*S			ITS4-ITS5		
Temprature	Time	Recurrent number	Temprature	Time	Recurrent number
80℃	4 min		94℃	4 min	
95℃	1 min		94℃	45 s	
50℃	1 min	32	50℃	1 min	32
65℃	1.5 min	Cycles	65℃	1.5 min	Cycles
65℃	5 min		65℃	7 min	
4℃	∞		4℃	∞	

Table S4. Variable sites from the cpDNA aligned sequences of *psb*AF-*trn*HR in the *Y. longistaminea*

Haplotyoe	*psb*AF-*trn*HR											
	4	16<br>9	176-18<br>1	20<br>8	258-27<br>3	28<br>4	33<br>8	33<br>9	34<br>0	341-34<br>4	34<br>5	346-34<br>7
Hap 1	A	C	①	G	②	A	T	T	T	③	C	TT
Hap 2	A	A	①	T	-	A	T	-	-	③	A	TT
Hap 3	-	A	-	T	-	A	T	-	-	③	A	TT
Hap 4	A	C	①	G	①	A	T	T	-	③	C	TT
Hap 5	A	A	①	T	-	A	-	-	-	-	-	--
Hap 6	A	A	①	T	-	A	-	-	-	③	C	TT
Hap 7	A	C	①	G	①	A	-	-	-	③	C	TT
Hap 8	A	A	①	T	-	C	-	-	-	③	A	TT

① ATTATT ②ATATATAAAATATAAA ③ATTT

Table S5. Variable sites from the cpDNA aligned sequences of *psb*AF-*trn*HR in the *Y. longistaminea*

Haplotyoe	*atp*B-*rbc*L									
	3	5	8	9	287	358-362	436	439	451-457	784
Hap 1	G	T	C	A	A	-	T	C	②	T
Hap 2	T	G	T	C	C	-	T	C	②	T
Hap 3	G	T	C	A	C	-	A	A	-	-
Hap 4	G	T	C	A	C	-	A	A	②	T
Hap 5	G	T	C	A	A	-	T	C	②	-
Hap 6	G	T	C	A	C	①	T	C	②	T
Hap 7	G	T	C	A	C	-	T	C	-	T

① ATTTA ②TTATATT

Table S6. Variable sites from the cpDNA aligned sequences of *trn*L-*trn*F in the *Y. longistaminea*

Haplotyoe	*trn*L-*trn*F														
	7	59	86-90	252	253-254	291	391	470	546	547	548	549	598	659	665
Hap 1	A	-	①	-	-	G	C	A	C	T	C	C	T	G	G
Hap 2	A	-	①	-	-	G	C	A	C	T	C	C	G	G	G
Hap 3	A	G	①	-	-	G	C	A	C	T	C	C	G	G	G
Hap 4	T	G	①	-	-	G	C	A	C	T	C	C	G	G	G
Hap 5	A	-	-	A	-	G	T	T	C	T	C	C	T	G	G
Hap 6	A	-	①	A	-	A	C	A	G	G	A	G	T	A	A
Hap 7	A	-	-	A	AA	G	C	A	C	T	C	C	T	G	G

① AATAA

Table S7. Variable sites from the cpDNA aligned sequences of *trn*G-*trn*S in the *Y. longistaminea*

Haplotyoe	*trn*G-*trn*S													
	23	66	91	171	279	280	281	282	283	284	285	405	411	476-482

Hap 1	C	T	G	C	-	-	-	-	-	-	A	A	T	-
Hap 2	C	T	A	C	-	A	T	A	T	A	T	A	T	-
Hap 3	C	T	G	A	T	A	T	T	A	T	A	A	T	①
Hap 4	A	T	G	C	-	-	-	-	-	-	A	A	T	-
Hap 5	C	G	G	C	-	-	-	-	-	-	A	G	G	-

① TATATAT

Table S8. Variable sites from the four cpDNA in the *Y. longistaminea*

Haplotyoe	cpDNA													
	3	5	8	9	287	358-362	436	439	451-457	784	983	990-995	1022	1075-1087
Hap 1	G	T	C	A	A	-	T	C	②	T	C	③	G	④
Hap 2	T	G	T	C	C	-	T	C	②	T	A	-	T	-
Hap 3	T	G	T	C	C	-	T	C	②	T	A	-	T	-
Hap 4	T	G	T	C	C	-	T	C	②	T	A	-	T	-
Hap 5	G	T	C	A	A	-	T	C	②	T	C	③	G	④
Hap 6	G	T	C	A	C	-	A	A	②	-	A	③	T	-
Hap 7	G	T	C	A	C	-	A	A	②	T	A	③	T	-
Hap 8	G	T	C	A	A	-	T	C	②	-	C	③	G	④
Hap 9	G	T	C	A	C	①	T	C	②	T	A	③	T	-
Hap10	G	T	C	A	C	①	T	C	②	T	C	③	G	④
Hap11	G	T	C	A	C	-	T	C	-	T	A	③	T	-

① ATTTA ② TTATATT ③ ATTATT ④ATATATAAAATATAAA

Haplotyoe	cpDNA													
	1098	1152	1153	1154	1155	1156-1159	1159	1160-1161	1221	1264	1289	1369	1477	1478-1482
Hap 1	A	T	T	T	A	TTT	C	TT	C	T	G	C	-	-
Hap 2	A	T	-	-	A	TTT	A	TT	C	T	A	C	-	①
Hap 3	A	T	-	-	A	TTT	A	TT	C	T	A	C	-	①
Hap 4	A	T	-	-	A	TTT	A	TT	C	T	A	C	-	①
Hap 5	A	T	T	-	A	TTT	C	TT	C	T	G	C	-	-
Hap 6	A	-	-	-	-	-	-	-	C	T	G	A	T	①
Hap 7	A	-	-	-	-	-	-	-	C	T	G	A	T	①
Hap 8	A	T	T	-	A	TTT	C	TT	C	T	G	C	-	-
Hap 9	A	-	-	-	A	TTT	C	TT	A	T	G	C	-	-
Hap10	A	-	-	-	A	TTT	C	TT	A	T	G	C	-	-
Hap11	C	-	-	-	A	TTT	A	TT	C	G	G	C	-	-

① ATATA

Haplotyoe	cpDNA													
	1098	1152	1153	1154	1155	1156-1159	1159	1160-1161	1221	1264	1289	1369	1477	1478-1482
Hap 1	A	T	T	T	A	TTT	C	TT	C	T	G	C	-	-
Hap 2	A	T	-	-	A	TTT	A	TT	C	T	A	C	-	①
Hap 3	A	T	-	-	A	TTT	A	TT	C	T	A	C	-	①
Hap 4	A	T	-	-	A	TTT	A	TT	C	T	A	C	-	①
Hap 5	A	T	T	-	A	TTT	C	TT	C	T	G	C	-	-

Hap 6	A	-	-	-	-	-	-	-	C	T	G	A	T	①
Hap 7	A	-	-	-	-	-	-	-	C	T	G	A	T	①
Hap 8	A	T	T	-	A	TTT	C	TT	C	T	G	C	-	-
Hap 9	A	-	-	-	A	TTT	C	TT	A	T	G	C	-	-
Hap10	A	-	-	-	A	TTT	C	TT	A	T	G	C	-	-
Hap11	C	-	-	-	A	TTT	A	TT	C	G	G	C	-	-

① ATATA

Haplotyoe	cpDNA											
	1483	1603	1609	1674-1680	1738	1790	1818-1822	1983	1984-1985	2022	2122	2201
Hap 1	A	A	T	-	A	-	②	-	-	G	C	A
Hap 2	T	A	T	-	A	-	②	-	-	G	C	A
Hap 3	T	A	T	-	A	G	②	-	-	G	C	A
Hap 4	T	A	T	-	T	G	②	-	-	G	C	A
Hap 5	A	A	T	-	A	-	②	-	-	G	C	A
Hap 6	A	A	T	①	A	-	-	A	-	G	T	T
Hap 7	A	A	T	①	A	-	-	A	-	G	T	T
Hap 8	A	A	T	-	A	-	②	-	-	G	C	A
Hap 9	A	A	T	-	A	-	②	A	-	A	C	A
Hap10	A	A	T	-	A	-	②	A	-	A	C	A
Hap11	A	G	G	-	A	-	-	A	AA	G	C	A

① TATATAT ②AATAA

Haplotyoe	cpDNA							
	2277	2278	2279	2280	2287-2294	2328	2390	2396
Hap 1	C	T	C	C	①	T	G	G
Hap 2	C	T	C	C	-	G	G	G
Hap 3	C	T	C	C	-	G	G	G
Hap 4	C	T	C	C	-	G	G	G
Hap 5	C	T	C	C	①	T	G	G
Hap 6	C	T	C	C	①	T	G	G
Hap 7	C	T	C	C	①	T	G	G
Hap 8	C	T	C	C	①	T	G	G
Hap 9	G	G	A	G	①	T	A	A
Hap10	G	G	A	G	①	T	A	A
Hap11	C	T	C	C	①	T	G	G

① CCTCAACT

Table S9. Variable sites from the rDNA aligned sequences of ITS4-ITS5 in the *Y. longistaminea*

Haplotyoe	ITS4-ITS5										
	8	115	158	193	364	431	480	526	527	628	632
Hap 1	G	A	T	A	C	C	A	T	-	-	-
Hap 2	A	A	T	A	C	C	A	T	-	-	-
Hap 3	G	A	T	A	C	C	A	T	-	-	C
Hap 4	A	A	T	A	C	C	A	T	-	-	C
Hap 5	G	A	C	A	T	C	G	T	-	-	C
Hap 6	G	A	T	G	T	C	G	T	-	-	C
Hap 7	G	A	T	A	T	C	G	T	-	-	C
Hap 8	G	G	T	A	C	C	A	T	-	-	C
Hap 9	G	A	T	A	C	A	A	T	-	-	C
Hap 10	G	G	T	A	C	C	A	T	-	G	C
Hap 11	G	A	T	A	C	C	A	T	-	G	C
Hap 12	A	G	T	A	C	C	A	T	-	-	C
Hap 13	G	A	T	G	C	C	A	T	-	-	C
Hap 14	-	A	T	A	C	C	A	T	-	-	C
Hap 15	G	A	T	G	C	C	A	T	T	-	C
Hap 16	G	A	T	G	C	C	G	C	T	-	C
