# Supplementary figures and images for: Unraveling the Evolutionary Tales of Yunnanopilia longistaminea (Opiliaceae): Insights from Genetic Diversity, Climate Adaptation, and Conservation Strategies
Source: Plants (Basel). 2025 Feb 26;14(5):706. doi: 10.3390/plants14050706 (PMC11901472; doi:10.3390/plants14050706)

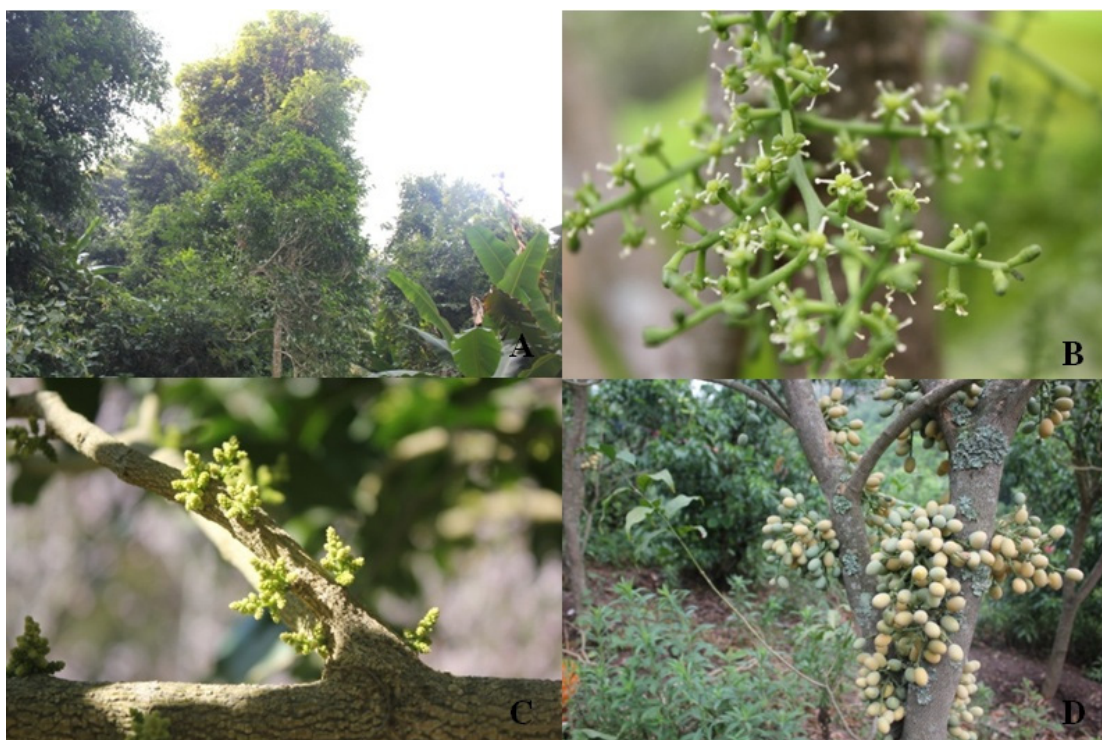

Figure S1 *Yunnanopilia longistaminea*: A. individual; B. leaves; C. florensence; D. friut

Supplement: Supplementary file 1 [file plants-14-00706-s001.zip › Supplementary Figure.pdf]
